# The role of short-term changes in cognitive capacity on economic expenditure among Kenyan agro-pastoralists

**DOI:** 10.1371/journal.pone.0247008

**Published:** 2021-03-03

**Authors:** Richard A. Iles, Aditi Surve, Samuel Kagundu, Haniel Gatumu

**Affiliations:** 1 School of Economic Sciences, Washington State University, Pullman, Washington, United States of America; 2 The Paul G. Allen School for Global Animal Health, Washington State University, Pullman, Washington, United States of America; 3 Compassion International—Kenya, Nairobi, Kenya; 4 Department of Psychology, University of Nairobi, Nairobi, Kenya; International Food Policy Research Institute, UNITED STATES

## Abstract

The increased exposure of pastoralist communities in East Africa to climatic shocks has focused attention on the resilience of these communities. Although many social scientists directly or indirectly infer versions of homo-economic agents, increasing evidence in development behavioral economics, indicates that such assumptions may be misplaced. Despite on-going advances in the science concerning the effects of stress on dynamic changes in short-term cognitive capacity, there remains limited understanding of the effects of changes in cognitive capacity on economic decision making. The present research empirically evaluates the drivers of short-term changes in cognitive capacity–cognitive ability and heuristic use–and its effect on crop and livestock expenditure among predominantly poor Kenyan agro-pastoralists. Three rounds of cognition and survey data from Samburu, Kenya is analysed. The primary data was collected at the end of the 2015–16 East African drought and covers an 11-month period between October 2016 and September 2017. Dynamic panel estimation, employing maximum likelihood, is used on balanced and unbalanced data. Results indicate that fluid intelligence and heuristic use, along with literacy and stressors, affect crop expenditure. Perceptions of scarcity, relative to prior expectations, are also identified as an important determinant of short-term changes in cognitive ability. These results underscore the importance of better understanding the effects of short-term changes in cognitive capacity on economic expenditure among the poor.

## Introduction

Eradication of poverty is a global priority. This priority is reflected in poverty eradication listed as the first Millennium Development Goal and Sustainable Development Goal. However, the barriers to poverty elimination are many. Barriers may be ecological or social, in line with socio-ecological systems (SES) [1, 2]. Sources of income for the rural poor are predominantly from the primary sector [3]. These earnings may be seasonal [4], irregular [5] and uncertain because of natural unforeseen contingencies (e.g. climate shocks such as drought, floods, cyclones, typhoons can ruin crops and kill livestock) [6]. Social barriers include those that are institutional [7, 8], or group-based [9, 10], while still others are internal to the individual [4, 11].

The economics literature concerning the existence of persistent poverty is vast. Much of this literature assumes that decision makers are fully rational. Bertrand et al. [12] highlight the frequent distinction made by social scientist between “highly rational” agents who “pursue their goals effectively, without mistakes, and with no need of help” (p.419). A contrast is then made with other researchers who attribute the poor with a “variety of psychological and attitudinal short-fallings” (p.419). Non-behavioral economic explanations of poverty relate to formal credit access barriers [13–15], poor human capital development [16, 17], and a lack of productive assets [6, 18]. A third-way, as proposed by Bertrand et al. [12], understands the effects of poverty on decision-making. This area of research is known as development behavioral economics.

The relationship between poverty and economic behavior is an expanding topic of research. Haushofer and Fehr [19], based on a review of the literature, conclude that stress and affect are likely channels through which poverty effects individuals’ risk preferences and time-discounting. More recent evidence further supports the existence of this likely casual pathway [20, 21]. Another aspect of economic behavior likely affected by poverty is cognitive capacity. The experimental evidence, from India and the US of Mullainathan and Shafir [22], and Huijsmans et al. [23], and the randomized control trial data of Mani et al. [4], links financial stress to short-term changes in cognitive capacity. Therefore, not only is the cognitive capacity of individuals less than full (i.e. limited by memory constraints, optimizing capacity, and subject to biases), but cognitive capacity is also dynamic in the short-run and is sensitive to real or perceived financial stress [24].

In the present context, cognitive capacity is defined in relation to reflective thought. Reflective thought is one of the two systems or domains of thinking that defines dual-processing theory [25, 26]. According to the Cognitive Reflection Test, cognitive ability (as measured by fluid intelligence) and heuristic use, are strong predictors of reflective thought [27, 28]. Therefore, cognitive capacity is, here, defined by the combined effects of cognitive ability and level of heuristic use.

Scarcity of resources can lower cognitive capacity. Traditionally in economics, scarcity is defined by constrained available resources to meet expanding demand. However, a scarcity mindset is defined by having a perception of not having enough [23, 29]. The scarce resources in question may be time, money, rainfall, etc. [22]. The perception of scarcity places a burden on cognitive capacity [22, 23]. Therefore, poverty may, not only involve meeting everyday expenses with less money, but also require the poor to do so with constrained cognitive capacity.

Cognitive ability is a latent and multidimensional concept [30]. In the economics literature, cognition is conceptualized as relating to inhibition (e.g. Flanker Task) [31], and executive control (e.g. numerical Stroop task) [27, 31]. As a measure of abstract reasoning of ‘new’ problems the use of fluid intelligence is also warranted. The seminal paper by Mani and colleagues [4] uses RPM to measure fluid intelligence, numerical Stroop task, and spatial compatibility task to measure the speed and accuracy of response. Several other economic studies also use RPM in Vietnam (middle income country) and the US and UK (high income countries) [20, 32, 33]. In contrast, working memory capacity (WMC) is a distinct construct frequently used in psychology to measure respondents’ ability to recall information in the face of distracting information [34–36]. However, it is not widely used in economics.

A growing literature identifies the links between cognitive capacity to decision making under uncertainty. Several lab-based experimental studies use fluid intelligence, as a measure of cognitive ability, and assess its impact on economic preferences [32, 37, 38]. Besedeš and colleagues use a choice experiment to identify the interaction between respondents’ age and their use of choice heuristics when making a health insurance plan choice [39]. While not directly controlled by Besedeš et al. [39], the tendency for fluid intelligence to decline with age suggests that these results reflect a relationship between fluid intelligence (i.e. cognitive ability) and level of heuristic use. These experimental results, taken in combination with the cognition and reflective thought literature [27, 28], indicate that cognitive capacity effects decision-making.

Global evidence of the effects of income shocks on cognitive capacity and economic decision-making are mixed. Carvalho et al. find that the effect of the regular pay cycle on executive function and memory and food expenditure in the US is negligible [31]. In Kenya, following a severe drought, the effect of increased fluid intelligence was positive on the decision to treat cattle for ticks [40]. Participants in this study were predominantly below the national rural poverty line. A study conducted in Vietnam among small business owners found that changes in risk preferences may be due to negative income shocks [20]. In the study by Dalton et al., small business retailers were presented with a series of hypothetical financial scenarios [20]. Retailers were randomly assigned into treatment (hard scenarios) and control groups (easy scenarios). Results showed that retailers in treatment group were less risk averse compared to those in the control group. This behavior was strongest for smaller shop owners and those who were not exposed to shocks involving large income volatility. Different measurement of cognitive capacity, the characteristics of income shocks sufficient to effect cognitive capacity, and the context of decision-making are all factors that may explain empirical differences.

As experimental evidence concerning the presence individual biases, dynamics of cognitive capacity and risk preferences has encompassed non-WEIRD (White, Educated, Industrialized, Rich and Democratic) samples [41], the generalizability of these results has strengthened. However, African agro-pastoralists, who frequently live in semi-arid environments, represent a unique population. Agro-pastoralists are financially dependent, to varying degrees, on livestock. Their livestock management practices are frequently characterized by high levels of spatial mobility [42]. Social and cultural practices that originate from economic dependence on livestock are also important [43]. Research by economists, anthropologists and ecologists concerning the livestock management behaviors of pastoralists is largely silent on the potential effects of a scarcity mindset on livestock management decisions.

Livestock management among agro-pastoralist is frequently considered from the assumption that decision makers are fully rational. Such fully rational decision makers are assumed to optimally use all available information to inform decision making [6, 44, 45]. This optimality is evident in the theoretical underpinning of “canonical neoclassical growth model” [6], and directly assumed producer rationality. Other social scientists, while being less explicit in their behavioral assumptions of pastoralists, suggest that pastoralists are optimizers. In the context of livestock reproduction Krätli and Schareike [46] argue pastoralists in Niger aim to maximize production, and not just reactively respond to harsh and marginal environments. Although the definition of constraints provided by Krätli and Schareike may differ from variables used by agricultural economists employing constrained optimization, the assumption of bounded rational, if not fully rational, agents is present:

“The Wodaabe do not bother with the attainment of ‘optimal’ production targets; rather, they exhibit a context-specific understanding of herd performance, where the measure of success is relative to competing fellow herdsmen and where the limit of success is open to continuous experimentation in ‘search of the better’”[46].

Such language conveys a sense that the referenced pastoralists could, “but don’t bother” to, seek to be optimizers. References to “search” and “limits” may reasonably be interpreted that these pastoralist agents are purposeful, seek to improve, and make complex production decisions with reference to well-being of other “herdsmen”. Such decision making requires cognitive capacity. Further claims about the decision-making of pastoralists are made, but with little, or no, testable supporting evidence [46, 47].

The present research empirically evaluates the drivers of short-term changes in cognitive capacity and their effects on crop and livestock expenditure among agro-pastoralists in central Kenya. Results indicate that fluid intelligence and heuristic use, along with literacy and stressors, affect crop expenditure and not livestock expenditure. A range of environmental and productive asset ownership measures are used to control for possible SES effects on expenditure decisions.

## Methods

Research design and associated tools used in the study were approved by the Washington State University Institutional Review Board (#16207) and the Kenyatta National Hospital-University of Nairobi Ethics and Research Committee (P613-10/2017). Oral consent was obtained from study participants.

In order to test the combined effects of cognition and heuristic use, as identified by the results of Cognitive Reflection Test of Frederick [27, 28], on livestock and crop expenditure, two measures of cognitive ability and one measure of heuristic are used. The measures of cognitive ability are fluid intelligence and working memory capacity. The short-form (20 out of the full 60 tasks) standard adult Raven’s Progressive Matrices (RPM) is used to measure fluid intelligence (see Fig A1 in S1 File), while working memory capacity is measured using a complex span Working Memory exercise (see Fig A2 in S1 File). The reliability of using 20 RPM tasks is established by Arthur and colleagues’ [48, 49] use of a 12 item advanced RPM test, and Bilker and colleagues’ [50] 9 item standard RPM test. No literacy and very limited numeracy is required to successfully complete both these tasks. This is particularly important in the context of adult Samburu agro-pastoralist. Although fluid intelligence is used in this study, no reference or interpretation is given to Intelligent Quota (IQ).

The relationship between fluid intelligence and working memory is debated. A meta-analysis conducted by Ackerman and colleagues [51], found that on average working memory tests correlated 0.364 with fluid intelligence. This average level of correlation is based on literate populations. At a latent level, strong correlation is predicted between fluid intelligence and working memory capacity [52]. RPM has been successfully used to study the Flynn effect among children in rural Kenya [53]. This was one of the first studies assessing the Flynn effect in a low-income country using RPM. The study by Daley et al. involved answering 60 tasks on the test which is the standard length of RPM test [53]. No known studies have used complex span counting tasks among East African populations.

Heuristic use is measured by the number of attributes ignored by respondents in a discrete choice experiment (DCE). Controlling for Attribute Non-Attendance (ANA) is common, within applied economics, as a means of controlling for respondents’ tendency to ignore product attributes [54–56]. The behavior of ignoring attributes, as a means of reducing the cognitive effort involved in comparisons, has a strong precedence in the psychology of decision making [57]. The choice context of the DCE, and the number of alternatives, attributes, and levels are detailed in the following Data section.

The estimated effect of perceptions of past income (Incomee) and loss of livestock are included in the analysis. Perceptions of financial well-being may play an important role in contributing to financial stress and changes in cognitive capacity. Early behavioral economic research by Katona [58] identified that perceived well-being during the recent past had a greater effect on cognitive capacity, compared to an equal change in forward looking perceptions. More recently, Ayllón and Fusco [24] identified a positive relationship between income and perceptions of financial stress. A range of factors may contribute to an expected asymmetrical relationship between forward and backward perceptions of financial well-being. These include: loss aversion [59], and the uncertainty of the future, and people are typically poor at estimating future returns. The livestock loss variable measures the loss of livestock relative to the current herd size. It controls for loss of livestock at the household level and provides a relative measure of the intensity of the loss. Among agro-pastoralist, the loss of livestock is expected to have financial and social capital losses [47] and therefore be associated with stress.

The relationship between cognitive capacity and its determinants is tested using both fixed effects and dynamic panel estimators. A Fixed Effects estimator is commonly used to identify the temporal effects of changes between variables. One strength of Fixed Effects estimation is that parameter estimates are unbiased in the presence of unobserved time invariant variables. Fixed Effect estimation controls for time variant variables, but is subject to the Nickell bias or the dynamic panel bias [60]. This bias occurs due to the correlation between the differenced regressor and the error term. The structure of fixed effects model is given by Eq (1).

Consider the following model with one independent variable. The subscripts *i* denotes individuals and *t* time periods. The parameter *u* represents the error term.

$$y_{it}-{\bar{y}}_{i}=\beta_{1}\left(x_{it}-{\bar{x}}_{i}\right)+\cdots +u_{it}-{\bar{y}}_{i}$$ (1)

An instrument variable approach may be used to correct for biased parameter estimates due to endogeneity. The existence of dual directional causation between measures of cognition and the perception of household financial well-being (Incomee) and recall of livestock lost is plausible. An instrument variable helps to control endogeneity because of the two characteristics it exhibits instrument erogeneity and instrument relevance. As per instrument erogeneity, the instrument variable should have no partial effect on dependent variable. According to instrument relevance, the instrument variable must be correlated with the endogenous explanatory variable. The use of lagged right-hand side variables may be used as an instrument. The correlation between the dependent variable and lagged variable reduces the further back a lag goes.

Dynamic panel-data estimators incorporate a first difference and instruments that are not correlated to the fixed effect. Generalized Method of Moments (GMM) systems are commonly used to overcome the dynamic panel bias, as specified by Arellano and Bond [61] and Roodman [62]. However, an alternate linear dynamic panel-data estimator is proposed by Moral-Benito, Allison and Williams [63]. This dynamic panel-data estimator uses maximum likelihood and may be used with balanced and unbalanced panel data. In the case of unbalanced panels, full information is used for missing data.

The linear Eqs (2) and (3) outline two baseline models estimated using a Fixed Effects. Fluid intelligence (RPM) and Working Memory Capacity (Count) are each used as dependent variables. The two equations differ by the inclusion of a lagged dependent cognition variable in Eq (3). Exogenous stressors, in Eqs (2) and (3), include the ratio of past livestock deaths relative to current livestock herd (as measured by FAO units) and rainfall. The use of the continuous variable ‘livestock loss’ provides a measure of the magnitude of households’ livestock loss, controlling for initial herd size. The exogenous effects of changes in rainfall is a natural stressor [21]. The study by Chemin et al. [64] identifies that the experience of drought among agriculturally dependent communities in Kenya causes increases in the stress hormone cortisol. Moreover, rainfall is associated with increased discounting and decreased investment in oxen and soil conservation among highland villagers in Ethiopia [21]. Estimates using Eqs (2) and (3) are based on a balanced panel.

$$Cognition=\beta_{0}+\beta_{1}.Incomee+\beta_{2}.Livestock\;loss+\beta_{3}.FAO+\beta_{4}.Rainfall+\beta_{5}.Fever+\beta_{6}.Ultra\_poor+\beta_{7}.Age+\varepsilon$$ (2)

$$Cognition=\beta_{0}+\beta_{1}.Cognition(lagged)+\beta_{2}.Incomee+\beta_{3}.Livestock\;loss+\beta_{4}.FAO+\beta_{5}.Rainfall+\beta_{6}.Fever+\beta_{7}.Ultra\_poor+\beta_{8}.Age+\varepsilon$$ (3)

Respondents’ perceived retrospective financial well-being, relative to prior expectations, is conceptualized as contributing a possible scarcity mindset. The control for prior expectations, which are often important in determining behavior [65, 66], enables the Incomee variable to control for respondent heterogeneity of personality trait and disposable income. The use of a binary measure enables estimation to account for the signal of expected household financial stress. The size of livestock holdings, as a measure of capital stock and wealth, is controlled by standardized measures of cattle (0.5), goats/sheep (0.1), camels (1.1) and oxen (0.5) using FAO units. Respondent health at the time of completing cognition tasks is controlled according to whether respondents had fever. Ultra-poor status represents an income poverty status, while age is an important determinant of cognitive ability.

The inclusion of the stressors in the above models represents an alternative modelling approach compared to the ‘before and after’ approach used by Carvalho et al. and Mani et al. [4, 31]. The Carvalho et al. study uses the regular pay cycle of respondents to induce stress. However, they don’t account for or control for prior expectations of household finances before and after pay day. The inclusion of ‘Incomee’ in Eqs (2) and (3) provides such a control. The randomization of harvesting time, as employed in the Mani et al. study, enables control for season effects, which are likely in an agricultural setting. The current study does not control for seasonal effects, however, the 11-month sampling period provides some indirect control for such effects.

The use of a maximum likelihood dynamic panel estimator (Moral-Benito et al., 2019) enables the inclusion of the non-varying binary variables. A third model is estimated. It includes no-schooling, in addition to the variables in Eq (3). Controlling for education level and age, in the context of low levels of basic education, is hypothesized important [67].

Eq (4) tests the effects of both cognition and heuristic use on crop and livestock expenditure using the maximum likelihood dynamic panel estimator. All three cognition variables are included when testing the determinants of household expenditure changes: fluid intelligence (RPM), working memory capacity (Count) and heuristics use (ANA). A one period lagged expenditure is also included. The same stressors, and controls for prior expectations, capital stock, health, no-schooling and poverty status are included, as per model 3. This model is used with the balanced and unbalanced panel data. The inclusion of an addition 325 respondents in the unbalanced panel increases the generalizability of results.

$$Expenditure=\beta_{0}+\beta_{1}.Expenditure(lagged)+\beta_{2}.ANA+\beta_{3}.RPM+\beta_{4}.Count+\beta_{5}.Incomee+\beta_{6}.Livestock\;loss+\beta_{7}.FAO+\beta_{8}.Rainfall+\beta_{9}.Fever+\beta_{10}.No\_school+\beta_{11}.Poverty\_status+\beta_{12}.Age+\varepsilon$$ (4)

### Data

The data for this study is obtained from a repeat cross-sectional survey carried out in central Kenya. The sample population is from Samburu county, Kenya. The Samburu are culturally related to the Maasai of East Africa. The county capital of Samburu, Maralal, is situated 350 kms north of Nairobi. The climatic environment is classified as semi-arid. The primary source of income of the sampled individuals comes from growing crops and raising livestock. Agro-pastoralism is a traditional form of occupation and lifestyle of the Samburu [68]. However, a range of policy and environmental changes are disincentivizing pastoralists to remain exclusively livestock dependent [43]. The practice of growing crops is increasingly frequent. The headcount poverty measure of Samburu was 75.8%, as per data collected between September 2015 and August 2016, with a poverty threshold of 3252 shillings [69].

The data was collected when the county was struck by a severe drought. Samburu typically receives rainfall twice a year- in March and April, and, October and November. Round 1 was conducted in the middle of the October-November rains in 2017, round 2 was before the March-April rains in 2018, and Round 3 was before the October-November rain in 2018. Over a 17-month period, from May 2017 to September 2018, rainfall was below the Long Term Average (LTA) in 13-months [70]. The first of these above LTA rainfall months was immediately before the first survey round, then immediately after the second round. During the six-months between rounds 2 and 3, three of these above LTA rainfall months occurred. However, the two months immediate prior to the third round experienced below LTA rainfall.

The sample size contains 401 surveyed individuals over a 11-month period. A total of 708 survey responses were given (see S1 File for study design description). A total of 168 participants (43%) completed one round of surveys (94 in round 1 and 74 in round 2), 156 participants (39%) completed two survey rounds (51 in rounds 1 and 2, 77 in rounds 2 and 3, and 28 in rounds 1 and 3), and 76 respondents completed three rounds (19%). Therefore, a total of 222 survey responses were given during round 1, 279 during round 2, and 182 during round 3. Table 1 presents descriptive statistics for the 76 respondents who completed all three survey rounds, and the 324 respondents who completed one and two survey rounds. The summary statistics are equivalent between the two groups. No evidence is present to suggest that the attrition is non-random in nature. A1 and A2 Tables in S1 File show the correlation structure of the variables, and results of a probit regression using panel equal to one as the dependent variable.

**Table 1:** Descriptive statistics.

Variable	Obs.	Mean/ Prop.	Stan. Dev.	Min.	Max.	Obs.	Mean/ Prop.	Stan. Dev.	Min.	Max.
	Panel (3 rounds)					Non-Panel (Single & 2 rounds)				
*Cognition*										
RPM–fluid intelligence	228	5.8	2.7	0	13	480	5.6	2.8	0	13
Count–Working Memory	228	7.1	5.6	0	39	480	7.0	7.1	0	55
*Heuristic*										
ANA	228	6.4	3.8	0	18	477	6.8	3.8	0	18
*Perceptions of Financial well-being*										
Perception of past income (Incomee)	228	0.9	0.3	0	1	476	0.9	0.3	0	1
*Socio-Economic*										
Non-poor	228	0.1	0.4	0	1	479	0.2	0.4	0	1
Regular Poor	228	0.3	0.5	0	1	479	0.3	0.5	0	1
Ultra-poor	228	0.5	0.5	0	1	479	0.5	0.5	0	1
Age (years)	228	41.9	14.7	18	79	479	42.9	14.5	18	82
No-school	228	0.6	0.5	0	1	480	0.7	0.5	0	1
*Livestock*										
Livestock units (FAO)	228	4.8	5.4	0	37	480	4.8	6.1	0	56
Ratio of livestock died relative to current herd (Stress)	228	74.7	79.3	-753	100	480	68.9	77.8	-900	100
*Environment*										
Rainfall (mm)	228	138.4	15.1	115	164	480	133.0	13.1	115	164
*Expenditure*										
Livestock	228	546.7	599.6	0	3200	479	719.8	1914	0	37500
Crops	227	821.0	888.9	0	6250	479	773.2	998	0	8750

The survey rounds coincide with reduced rainfall. Because the sampled individuals belong to agro-pastoral communities, lack of rainfall has a direct impact on their earnings and becomes a significant source of stress.

**Fig 1:**
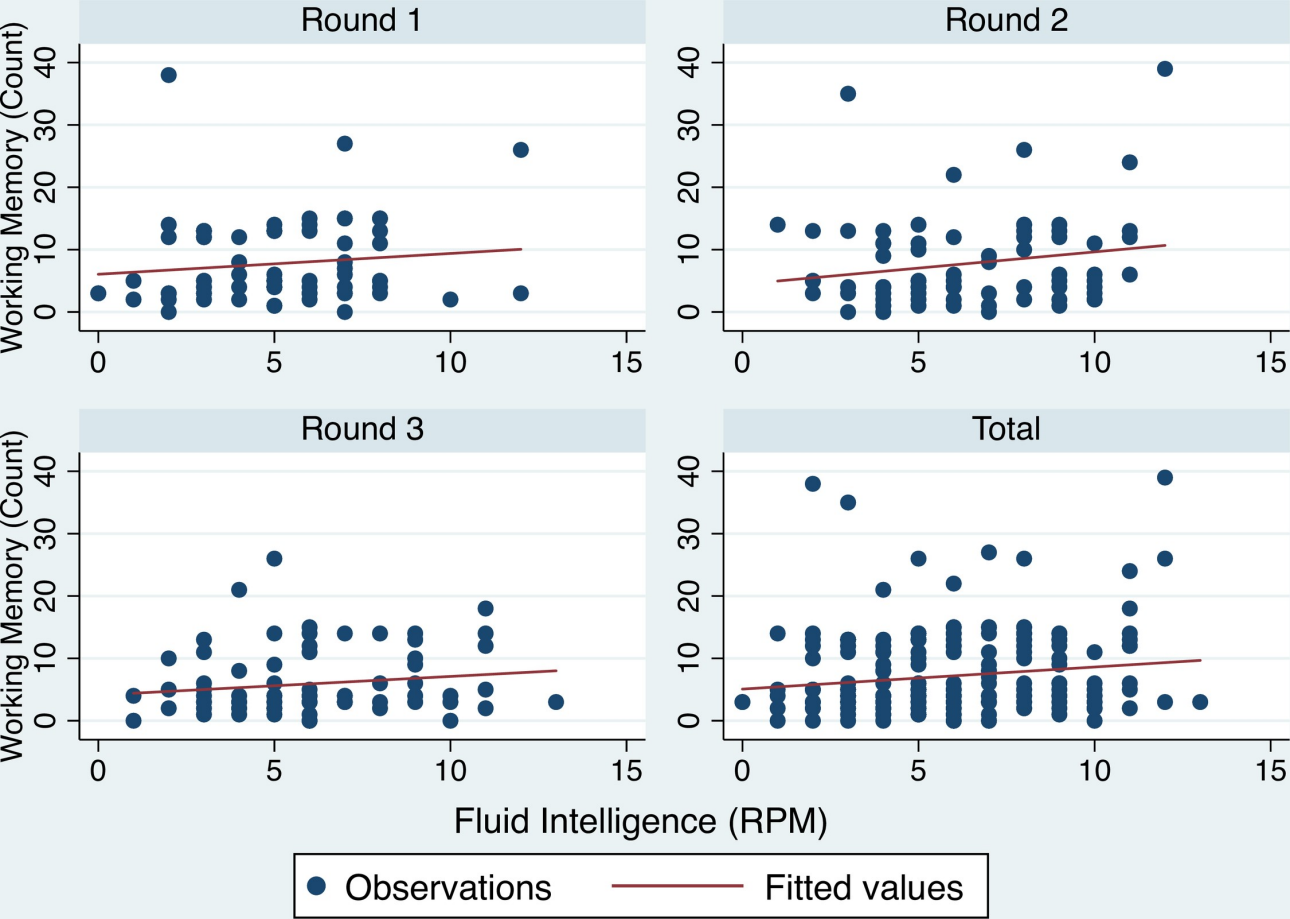
Count and RPM scores by round. Scatter plot of Count and RPM scores by survey round.

The summary statistics for RPM are found to be similar in magnitude compared to the Daley et al. study [53]. The ratio of mean RPM score to tasks completed in the Daley et al. study was 12.82 /60 = 0.21. This is very close to current study 5.8/20 = 0.29 (Table 1). The analysis of the determinants of WMC (Count) uses scores ranging between 0 and 55. The complex span Working Memory Capacity task was adaptive. Respondents who answered correctly were given more tasks to answer. The Count means and standard deviations are 7.1 and 7.0, and 5.6 and 7.1. Fig 1 presents a scatter plot of Count and RPM across each survey round.

**Fig 2:**
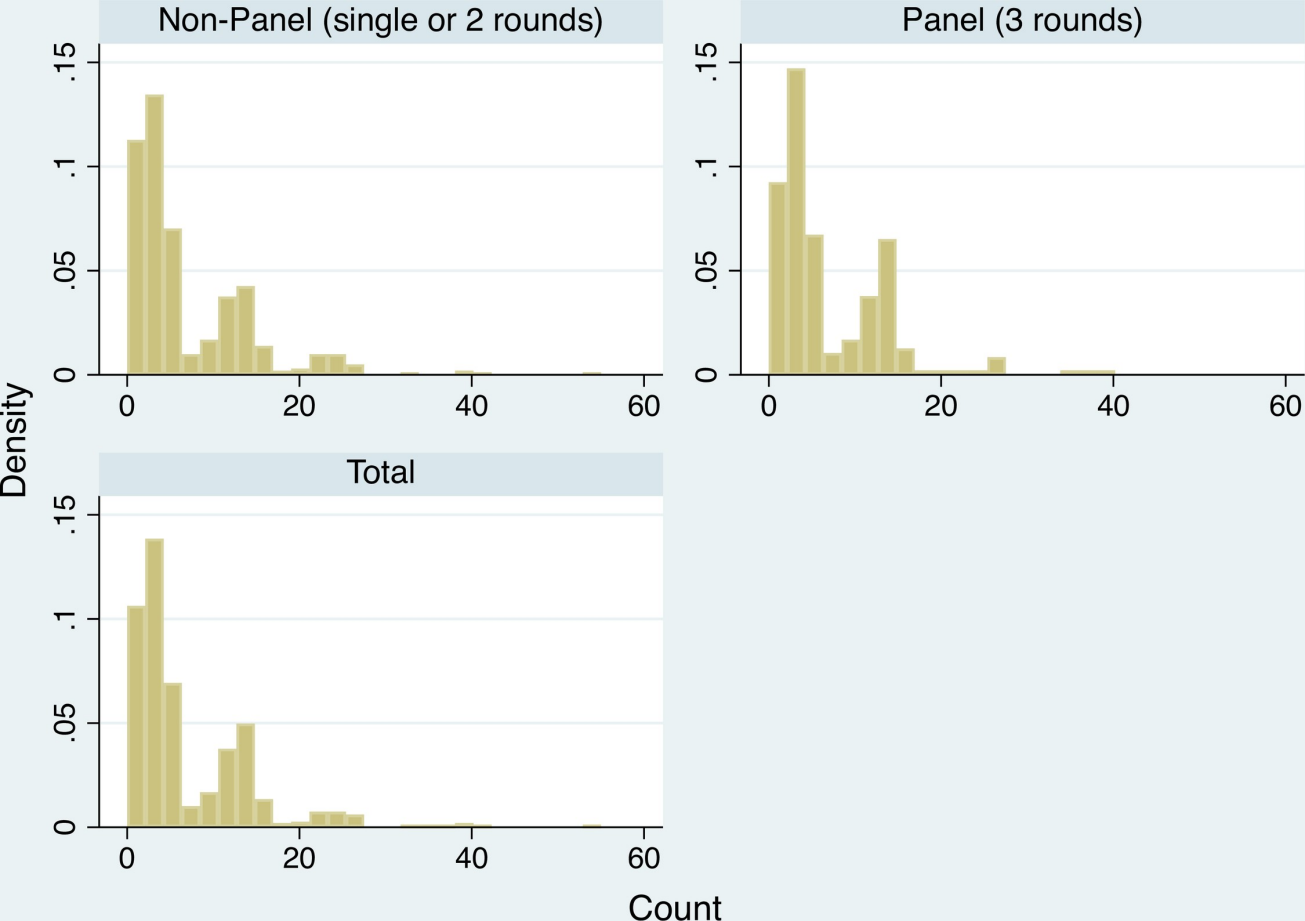
Count scores–panel vs non-panel. Histogram (density) of Count scores.

**Fig 3:**
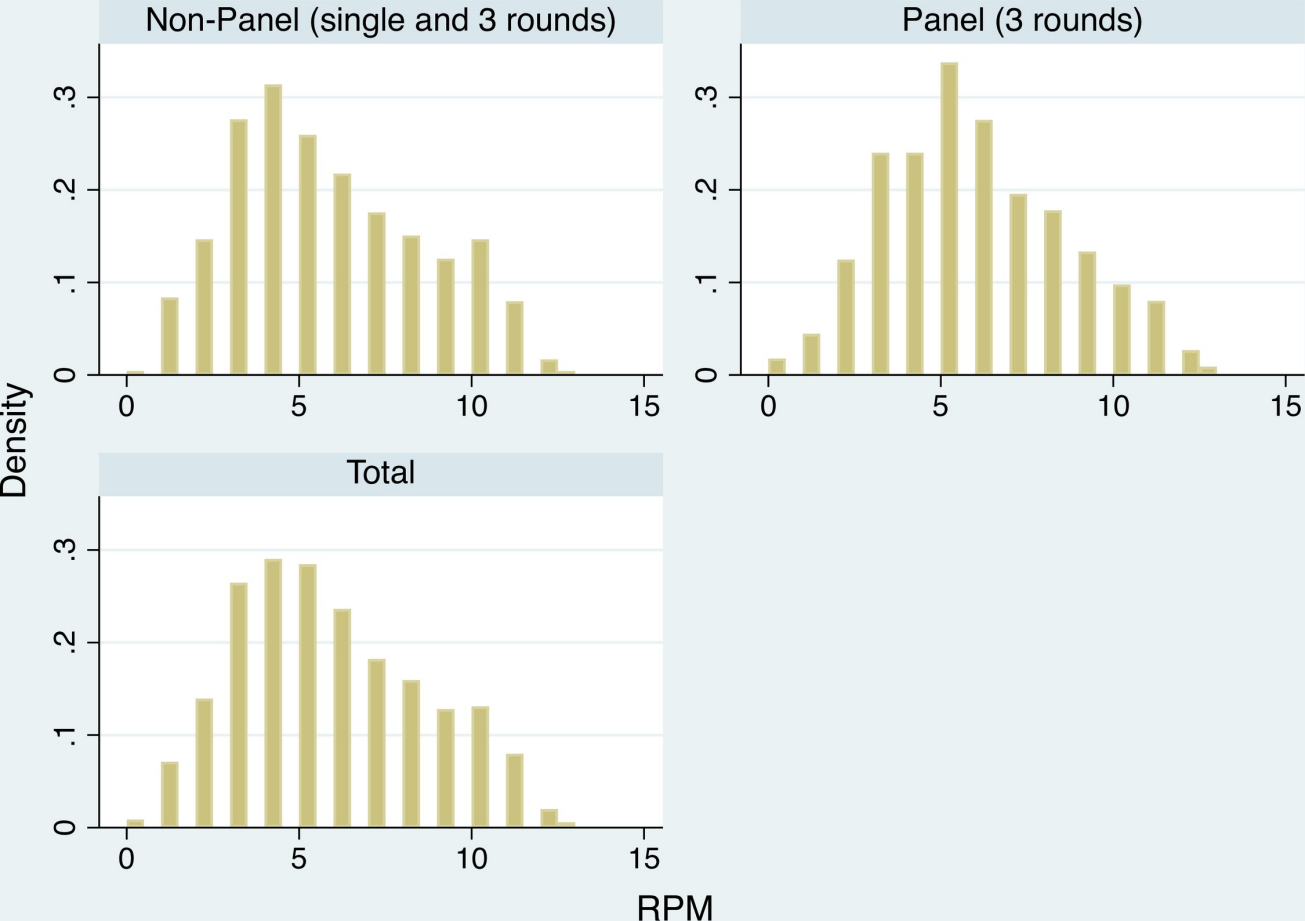
RPM scores–panel vs non-panel. Histogram (density) of RPM scores.

The histograms of the Count and RPM variables indicate little difference exists in the distribution of the respective scores between respondents in the 3-round panel and those not. Fig 2 plots the density of Count scores for each group, plus a combined total. The distributions are bi-modal, with a mode at ~6 and another at ~16. Fig 3 compares the distributions for the variable RPM for the panel and non-panel cohorts.

The Pearson correlation between each measure of short-term cognitive capacity is 0.15. This holds for both cohorts (balanced panel vs non-panel). The correlation, for the balanced panel respondents, between RPM and Count was 0.124 in Round 1, 0.189 in Round 2, and 0.149 in Round 3. The absence of serial increases in the correlation suggests that there was no systematic learning across repeated cognition measure. The sub-set of 20 RPM tasks used in each survey round varied. The, at least, five-month duration between survey rounds also helped prevent against systematic learning. The relatively low correlation scores indicate that working memory and fluid intelligence measure distinct unobserved cognitive dynamics.

The Discrete Choice Experiment (DCE) used was unlabeled with three choice alternatives related to a decision to vaccinate livestock against Contagious Bovine Pleuropneumonia (CBPP) [71]. Each alternative was defined by four attributes. These were: price (4 levels), distance to access vaccine (4 levels), disease risk information received (3 levels), and likelihood of vaccine side-effects (4 levels). Respondents completed 6 separate choice tasks. Therefore, the maximum ANA count was 18 (3x6). CBPP is a livestock disease well understood by respondents. The disease is perceived as a major threat in East Africa [72]. The mean ANA count for each respondents was 6.66.

The Pearson correlations between ANA and each cognition measures (RPM and Count) are equivalent in magnitude and negatively signed. The correlation between RPM and ANA is -0.047 (for panel respondents) and -0.078 (for non-panel respondents). Equivalent measures are evident for the correlation between Count and ANA (-0.074 and -0.005). This negative sign reflects the expected relationship between cognitive ability and use of a heuristic. Although the strength of the negative relationship is weak and not statistically different from zero.

**Fig 4:**
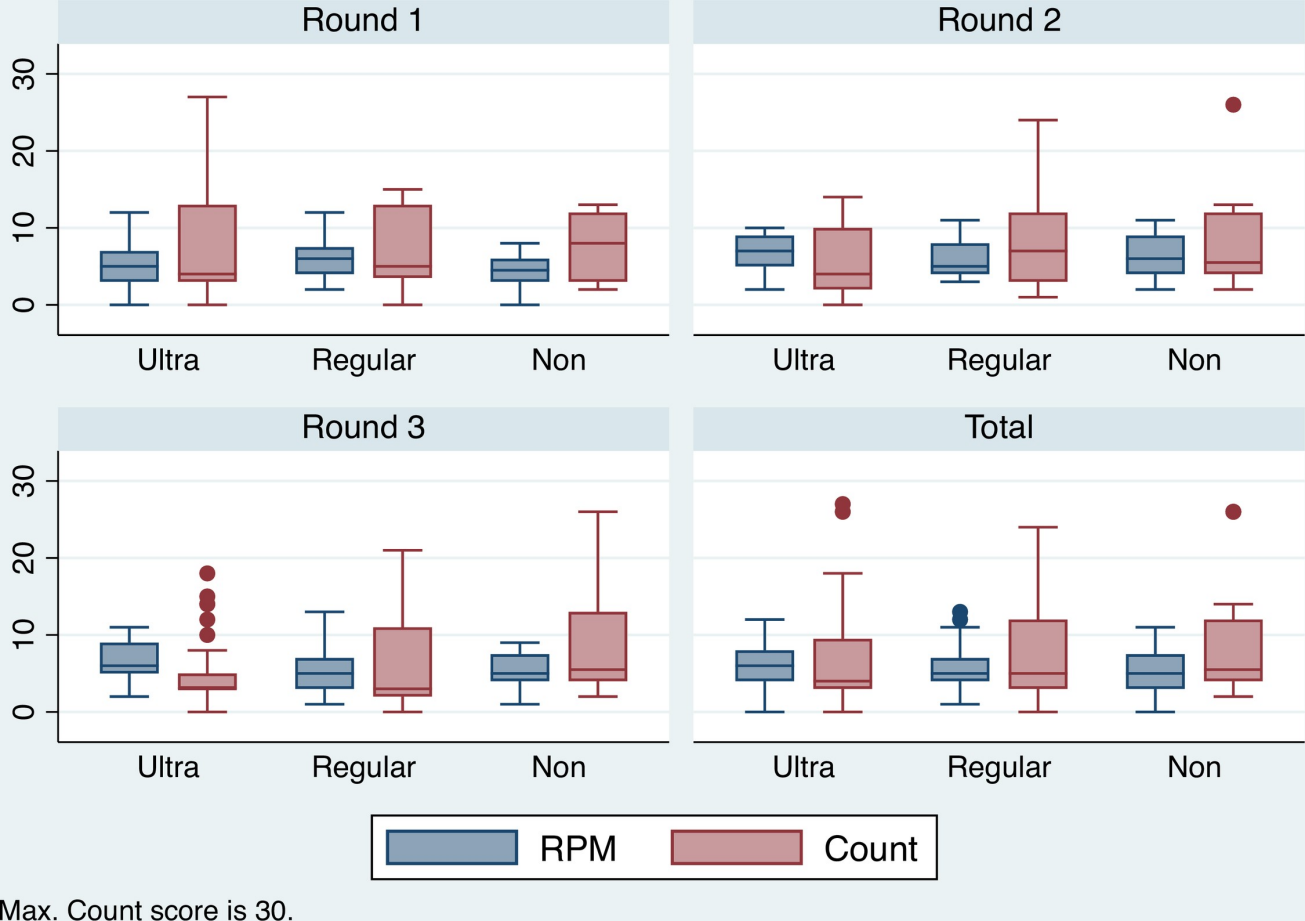
Distribution of RPM and Count scores by poverty level. Boxplot showing the relationship between RPM, Count and poverty levels, across survey rounds (Count max. 30).

Non-poor, regular-poor, and ultra-poor are binary variables measuring degrees of income poverty. Non-poor accounts for respondents with monthly income above 3252 Kenyan shillings per person. Regular-poor accounts for respondents with monthly income between 1000 and 3252 shillings. Remaining respondents belong to ultra-poor with monthly income less than 1000 shillings per month. Household income is calculated by estimating summing monthly income streams from livestock, crops, and salaries/wages. It does not include remittances or in-kind transfers. Table 1 shows the proportion of respondents falling into each income category. Fig 4 presents a series of boxplots demonstrating the descriptive relationship between cognitive ability across the three income categories. From the interquartile range there appears no meaningful difference in the absolute cognition scores across income groups. It is important to highlight the fact that panel estimators used in this analysis are not concerned with absolute levels of cognition, but changes.

**Fig 5:**
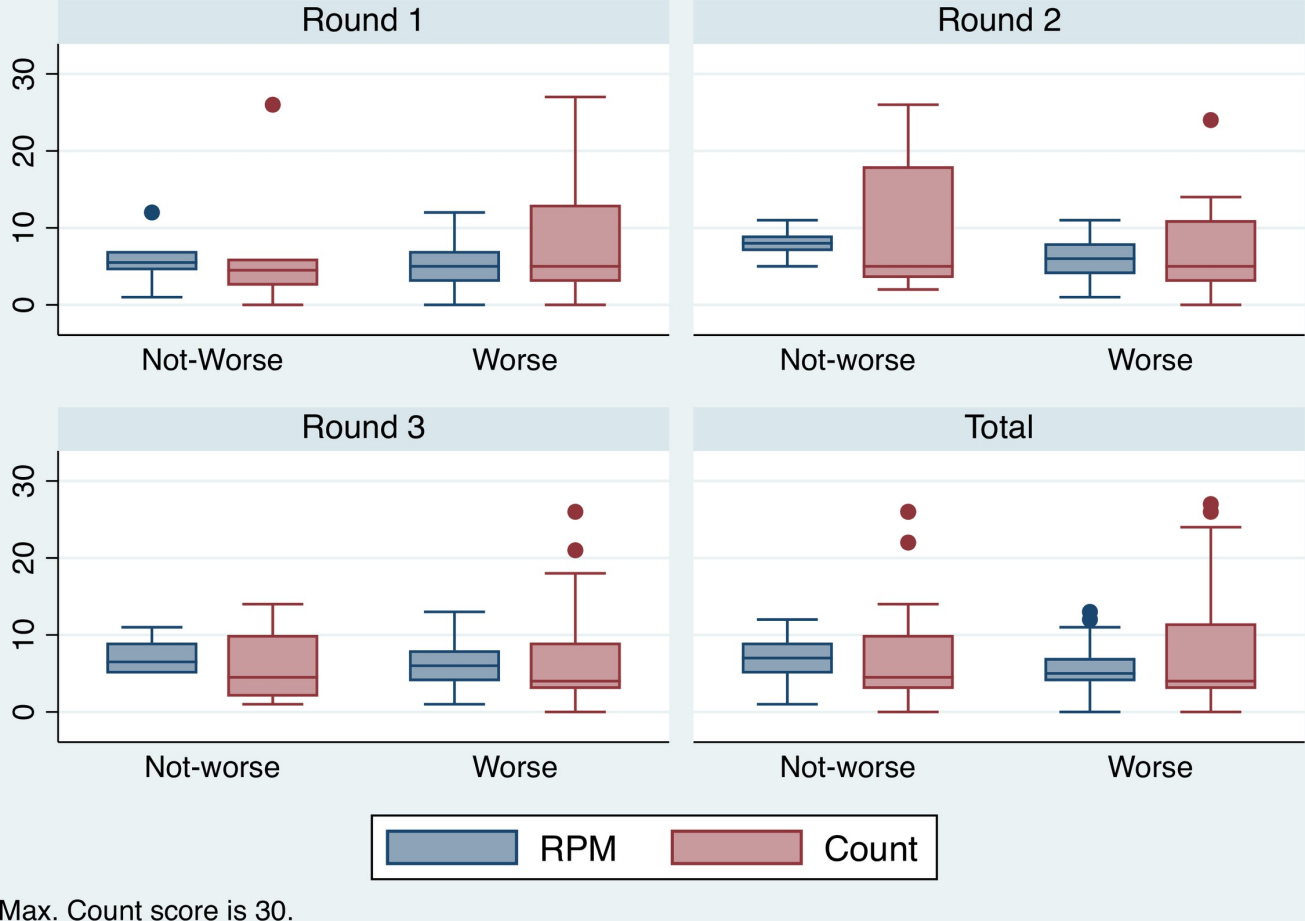
Distribution of RPM and Count by perception of past income. Boxplot showing the relationship between RPM, Count and perception of past income (1 = worse than expected) across three survey rounds (Count max.30).

The variable of perception of past income (Incomee) is a binary variable that measures whether respondents perceived their household finances were worse than expected. Fig 5 presents a series of boxplots showing the relationship between the RPM, Count and Incomee. The mean and interquartile ranges of Count, for each level of prior expectation of household finance, shows no systematic difference, either within or between rounds. The mean RMP scores is higher in the ‘Not-worse’ category compared to the ‘Worse’ category within each survey round.

Age is measured in years with a mean respondent age of 41.9 years in the panel cohort and 42.9 years for the non-panel cohort. The ‘no-school’ measure is binary and measures the proportion of respondents who self-reported as having no schooling. The level of no-schooling among the panel cohort is less than compared to the non-panel cohort at the five percent level of significance (two sample proportions-test that the difference is not zero: p-value of 0.028). Among the panel cohort the proportion of respondents self-reporting no schooling is 0.61, while the corresponding level among the non-panel cohort is 0.69. This difference is a function, in part, of the slightly older respondent sample in the unbalanced panel. Those who are above the age of 50 are more likely to report that they have no-schooling.

The Food and Agriculture Organization standardized measure of livestock units is used to aggregate the number of cattle (0.5), goats (0.1), camels (1.1) and oxen (0.5) owned by each household. The variable livestock loss is a measure of the ratio of livestock that died in the recent past relative to the current herd size multiplied by 100. Rainfall (mm) data is based on accumulated monthly data and is based on a 1 km squared area [73]. There is a difference between the mean rainfall of the balanced panel compared to the unbalanced. The mean monthly rainfall for the balanced panel is 138.4 mm, while for the unbalanced panel it is 133.0 mm. This difference is statistically significant at the one percent level. This difference is a result of the differing proportion of respondents from each of the five sample communities in the balanced and unbalanced panels.

The mean expenditure on crops between the balanced and unbalanced panels is not statistically different. The balanced panel has a mean of KES 821.0 and the unbalanced mean is KES 773.2. There is a difference in the mean livestock expenditure between the two panel groups (KES 546.7 vs KES 719.8). This difference is expected to affect comparisons of livestock expenditure between the balanced and unbalanced panels.

## Results

Parameter estimates using a Fixed Effects estimator are presented in Table 2. Model 1.0 contains no lagged dependent variables. The variables of Incomee and Livestock loss are both identified as negatively associated with RPM at the one percent level of significant. The inclusion of the one period lagged RPM variable, in model 2.0, eliminates the statistical significance of the Livestock loss variable. The magnitude of the Incomee variable in model 2.0 is reduced (absolute value) and the level of statistical significance is reduced to five percent. The one period lagged RPM dependent variable is highly significant. Controlling for respondents’ RPM scores between rounds 1 and 2, is predicted to have a strong negative predictive value on the change in scores between rounds 2 and 3.

**Table 2:** Relationship between RPM and determinants.

	Model 1.0			Model 2.0			Model 3.0		
	Coeff.		Std. Err	Coeff.		Std. Err	Coeff.		Std. Err
RPM (lagged)	-		-	-0.614	***	0.102	-0.345	*	0.161
Incomee	-1.749	**	0.584	-1.039		0.617	-1.413	*	0.635
Livestock loss	-0.009	***	0.002	0.002		0.003	-0.001		0.004
FAO units	-0.070	**	0.029	0.005		0.056	-0.021		0.055
Rainfall	0.029	**	0.010	0.013		0.012	0.031		0.036
Fever	0.266		0.537	-0.415		0.573	-0.198		0.580
Ultra-poor	0.279		0.375	0.353		0.454	0.310		0.452
Age	0.052		0.051	0.096		0.060	0.077		0.067
No-schooling	-		-	-		-	-2.230	*	1.003
Constant	1.999		2.519	4.490		2.867	-		-
N (n)	76 (228)			76 (152)			76 (152)		
AIC	917			488			6198		
BIC	941			512			6373		
F-stat (p-value)	6.8 (<0.001)			9.2 (<0.001)			-		
Chi^2^ (p-value)	-			-			29.1 (<0.001)		

Note: Statistical significance 0.05 level denoted by

*, 0.01 level denoted by

**, and 0.001 level denoted by

***. N represents the number of respondents and n represents number of observations. Robust standard errors used (clustered by individual in models 1.0 and 2.0).

The model 3.0 output, in Table 2, extends model 2.0 by including the binary variable–no-school. The inclusion of this variable, plus the use of the dynamic panel estimator, reduces the magnitude of the lagged RPM term. In model 3.0 the lagged term remains negative, but is statistically significant at the five percent only. The predicted effect of no-schooling on RPM is negative and statistically significant at the five percent level. Experiencing worse household finances than previously expected is predicted to have a negative effect on RPM scores.

The output in Table 3 replicates the models presented in Table 2, but with Count as the dependent variable. Only changes in Rainfall are predicted to negatively effect changes in Count in model 1.1. The inclusion of the one period lagged Count variable in model 2.1 is predicted to have a strong negative effect on changes in current Count scores. The predicted negative effect of Rainfall on Count remains statistically significant at the one percent level. Due to convergence difficulties using the dynamic panel (maximum likelihood) estimator, the one period lagged dependent variable is omitted. Therefore, model 3.1 extends model 1.1 by including the binary variable of no-school. The only variable found to have a statistically significant effect on Count, in model 3.1, is no-schooling.

**Table 3:** Relationship between Complex Span Counting task and determinants.

	Model 1.1			Model 2.1			Model 3.1		
	Coeff.		Std. Err	Coeff.		Std. Err	Coeff.		Std. Err
Count (lagged)	-		-	-0.584	***	0.136	-		-
Incomee	-0.406		1.900	0.976		1.790	-0.133		1.883
Livestock loss	-0.009		0.014	-0.003		0.007	-0.008		0.014
FAO units	0.034		0.071	0.159		0.136	0.045		0.077
Rain	-0.076	**	0.025	-0.089	*	0.034	-0.063		0.101
Fever	-1.890		1.060	-1.656		1.391	-1.852		1.059
Ultra-poor	-1.691		0.906	-2.028		1.097	-1.867		0.985
Age	0.021		0.121	-0.046		0.122	0.009		0.120
No-schooling	-		-	-		-	-3.027	*	1.480
Constant	19.120	**	7.167	26.168	***	7.017	-		-
N (n)	76 (228)			76 (152)			76 (152)		
AIC	1345			805			8263		
BIC	1369			829			8438		
F stat (p-value)	2.2 (0.043)			6.5 (<0.001)			-		
Chi^2^ (p-value)	-			-			20.1 (0.010)		

Note: Statistical significance 0.05 level denoted by

*, 0.01 level denoted by

**, and 0.001 level denoted by

***. N represents the number of respondents and n represents number of observations. Robust standard errors used (clustered by individual in models 1.1 and 2.1).

Table 4 presents the dynamic panel estimates of i) cognition on heuristic use, and ii) cognition and heuristic use on crop expenditure. The first set of parameter estimates in Table 4 (ANA as dependent variable) indicate that only RPM is a good predictor of changes in ANA heuristic use. No-schooling is also not a good predictor, nor is the one period lagged dependent variable. Convergence problems prevent the inclusion of Incomee, Livestock loss, and Fever. The subsequent two model outputs, presented in Table 4, each have crop expenditure as the dependent variable. The first of these parameter estimates uses the balanced panel dataset. Using the balanced panel data, heuristic use, rainfall and age are estimated to have a negative effect on crop expenditure, each at the five percent level. In addition to these statistically significant parameter estimates, RPM, FAO units, and Ultra-poor status are predicted to be statistically significant when uses the unbalanced dataset. In contrast to the predicted role of cognition and heuristic use with respect to crop expenditure, the same variables are estimated to have no statistically significant effect in predicting livestock expenditure.

**Table 4:** Heuristic use and crop expenditure–balanced and unbalanced datasets.

	ANA (balance)			Expend–Crops (balanced)			Expend–Crops (unbalanced)		
	Coeff.		Std. Err	Coeff.		Std. Err	Coeff.		Std. Err
Dependent (lagged)	0.101		0.124	0.306		0.306	-0.186		0.171
ANA	-		-	-69.110	*	32.152	-47.286	**	15.343
RPM	0.332	*	0.155	-77.162		75.613	-94.603	**	33.504
Count	-0.015		0.054	6.736		14.426	2.452		11.956
Incomee	-		-	553.917		346.965	264.952		177.449
Livestock loss	-		-	-1.270		1.239	0.229		0.972
FAO units	-0.089		0.122	-40.873		32.563	-40.621	*	19.405
Rain	0.024		0.061	-35.678	*	18.176	-11.764		9.690
Fever	0.161		0.777	-64.647		315.244	3.740		156.559
Ultra-poor	-		-	-487.755		261.093	-294.981	*	118.925
Non-poor	0.185		1.131	-		-	-		-
Age	-0.021		0.088	-68.914	*	32.043	-26.726	*	15.508
No-schooling	-0.053		0.853	403.480		400.664	-129.428		191.857
N (n)	76 (152)			76 (152)			400 (708)		
AIC	6226			11012			35556		
BIC	6401			11183			36366		
Chi^2^ (p-value)	10.3 (0.327)			42.0 (<0.001)			43.9 (<0.001)		

Note: Statistical significance 0.05 level denoted by

*, 0.01 level denoted by

**, and 0.001 level denoted by

***. N represents the number of respondents and n represents number of observations. Robust standard errors used.

Table 5 estimates the same models, as used in Table 4, with livestock expenditure as the dependent variable. Neither of the cognitive ability variables, nor the heuristic use variable, are estimated to predict household livestock expenditure. The perception of household finances relative to prior expectations (Incomee) is estimated to positively predict livestock expenditure, at the one percent level, using the balanced panel data. The parameter magnitude and level of significance diminishes when using the unbalanced dataset. Age has a negative effect on livestock expenditure across both datasets. Not being poor is predicted to have a positive effect on expenditure.

**Table 5:** Livestock expenditure–balanced and unbalanced datasets.

	Expend–Livestock (balanced)			Expend–Livestock (unbalanced)		
	Coeff.		Std. Err	Coeff.		Std. Err
Dependent (lagged)	-0.028		0.124	-0.298	*	0.124
ANA	-20.240		19.263	-24.978		19.905
RPM	16.255		25.372	-18.672		32.640
Count	-0.543		8.961	2.349		7.451
Incomee	488.247	***	137.673	113.934		213.366
Livestock loss	-0.865		0.601	-1.169		0.637
FAO units	-0.570		24.116	3.220		20.737
Rain	1.152		9.910	8.646		8.811
Fever	-11.411		205.522	297.200		192.634
Non-poor	174.786		151.398	252.091	*	128.746
Age	-27.786	*	13.464	-9.817	*	15.616
No-schooling	328.506		185.857	-72.477		169.605
N (n)	76 (152)			400 (708)		
AIC	10953			35396		
BIC	11128			35863		
Chi^2^ (p-value)	37.5 (<0.001)			21.7 (0.041)		

Note: Statistical significance 0.05 level denoted by

*, 0.01 level denoted by

**, and 0.001 level denoted by

***. N represents the number of respondents and n represents number of observations. Robust standard errors used.

## Discussion

A comparison between the Fixed Effects are the dynamic panel (maximum likelihood) model estimates reveals that the added flexibility of including the time invariant variable–no-school–is important. Models 3.0 and 3.1 both include this variable. The dynamic panel estimates are negative and statistically significant for no-schooling as a predictor of changes in fluid intelligence and working memory capacity measures. This result supports the contention that cognitive ability is, in part, a function of education. The importance of hypothesized stressors in predicting changes in cognitive ability indicates that the measures of fluid intelligence and working memory capacity remain meaningful for the given population. The consistency of statistically significant parameter estimates across models 1.0–3.0, such as ‘Incomee’, and ‘Rainfall’ between models 1.1–3.1, supports the robustness of the dynamic panel (maximum likelihood) estimator.

The inclusion of lagged cognitive ability dependent variables is a strong predictor of current cognitive ability. The importance of including lagged dependent cognitive ability variables is underscored by the parameter estimates for models 2 and model 3.0. The lack of convergence when including the lagged Count variable prevented its inclusion in model 3.1. In all models, where it was included, the relevant coefficients are negative. The inclusion of the lagged cognitive ability variable is a control for long-term cognitive ability. As such, its inclusion helps to identify stressors and demographic characteristics that affect short-term changes in cognitive ability.

The above models provide evidence that exogenous and endogenous stressors affect short-run cognitive ability, but not heuristic use. Rainfall is consistently predicted an important determinant of WMC (as measured by the Count variable). While the perceived household financial status, relative to prior expectations, has a predicted negative effect on fluid intelligence (as measured by short-form RPM). The importance of household perceptions of household finances, relative to prior expectations, on short-term fluid intelligence supports the scarcity thesis that perception of scarcity places a tax on cognitive capacity.

Evidence that fluid intelligence and WMC are both effected by stressors indicates that WMC measures of cognitive ability should be considered by future studies. The distinction between heuristic use and cognitive ability, when defining cognitive capacity, is underscored by the fact that no stressors were found to affect ANA.

The distinction between determinants of changes in heuristic use and cognitive ability is seemingly contradicted by the fact that fluid intelligence is a positive predictors of changes in ANA. The dual features of independence and interdependence of heuristic use and cognitive ability, as identified in this study, is consistent with the experimental identification of reflective thought [27, 28].

Crop expenditure is affected by a mix of cognitive capacity, external conditions and respondent age. These results are robust to use of the balanced and unbalanced datasets. The negative coefficients for ANA and RPM indicate opposing cognitive capacity effects. On the one hand, as heuristic use increases crop expenditure is predicted to decrease. A priori we expect that increases in ANA would be associated with a greater likelihood of ‘fast-thinking’ (i.e. System 1—dual processing theory). In many cases such ‘fast thinking’ is associated with sub-optimal decision-making in the context of uncertain outcomes. In the context of crop expenditure following a prolonged and severe drought, the degree of uncertainty associated with crop investments is likely greater. As such, we believe that the negative parameter estimate for ANA is as expected.

On the other hand, increasing fluid intelligence is predicted to lower crop expenditure. A priori the expected sign of RPM is indeterminate. Given the environmental context, whether higher levels of fluid intelligence are necessarily associated with positive or negative investments in uncertain. The negative coefficient for livestock holdings (FAO units) suggests that agro-pastoralist households use crop income as a substitute to livestock income. With decreasing herd sizes, some households may be feel compelled to invest in crops following a severe drought, even if they know that yields may be limited. The effects of changes in cognitive capacity on Kenyan agro-pastoralists’ crop expenditure suggests that farmers’ decision-making is not consistent. If so, this stands in contrast to the assumptions of Duflo and colleagues fertilizer model [74], but provides some support to the relationship between impatience and fluid intelligence [75].

The absence of a predicted effect of cognitive capacity on livestock expenditure is noteworthy. Possible explanations for the absence of effects on livestock expenditure include: the nature of livestock expenditure at the end of a severe drought may not be susceptible to the effects of changes in cognitive capacity; or Kenyan agro-pastoralists have developed livestock management practices that are immune to changes in cognitive capacity [46, 76]. The deep cultural knowledge concerning livestock management among the Samburu, or location-related fixed effects (i.e. access to schools), are likely explanation for the estimated absence of no-schooling affecting livestock expenditure.

The interpretation of results is limited in important ways. The short nature of the panel limits the number of dependent variable lags available. As a result, endogeneity biases may be only weakly controlled. Potential seasonal effects are not fully controlled. Furthermore, the ability to generalize results beyond Kenyan agro-pastoralists is limited. The specific environmental effects, as financial stressors for Kenyan agro-pastoralist, may not be replicated in other contexts. However, the results taken in conjunction with results from other low and high income contexts suggests that the results are consistent with the developing literature.

## Conclusion

The identified drivers of short-term changes in cognitive capacity among agro-pastoralist include exogenous environment stressors and endogenous perceptions of financial well-being. Scarcity is shown as an important determinant of cognitive ability. The effect of perceptions of financial well-being on cognitive ability is further evidence of the importance of perceptions of scarcity on cognitive performance. The effect of changes in rainfall, as a stressor, on cognitive ability also confirms the importance of exogenous stressors. Although these results are interpreted within the context of Kenyan agro-pastoralist communities, there exists reason to believe that these drivers may also affect other agricultural communities.

The contrasting effects on cognitive capacity–cognitive ability and heuristic use–on crop expenditure provides evidence that scarcity and perception of scarcity effects economic decision making. While such results are subject to seasonal effects, the results presented identify a likely relationship between scarcity and economic decision making.

## Supporting information

S1 File (DOCX)
