# Supplementary material for: The role of short-term changes in cognitive capacity on economic expenditure among Kenyan agro-pastoralists
Source: PLoS One. 2021 Mar 3;16(3):e0247008. doi: 10.1371/journal.pone.0247008 (PMC7928487; doi:10.1371/journal.pone.0247008)
Supplement: S1 File — (DOCX) [file pone.0247008.s001.docx]

**Supplemental Material**

**
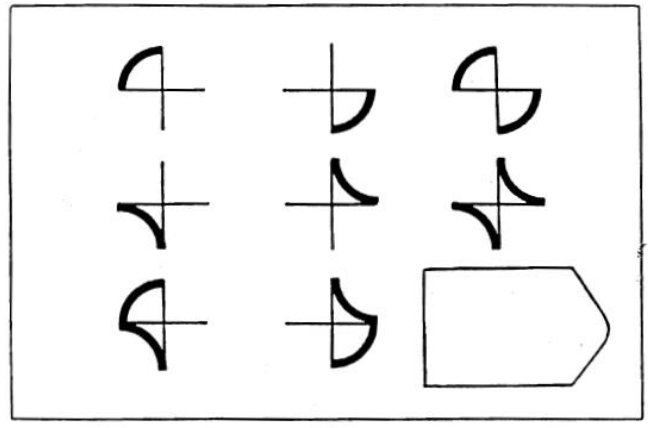
**

**Figure A1: Example of a Raven’s Progressive Matrix task.**

The relationship between two constructs of cognition and economic decision-making is tested in the current study by using measures of fluid intelligence and Working Memory Capacity (WMC). Both measures require no minimum level of literacy to understand or complete. A short form standard RPM task—*gf*—and an adaptive Counting Span task—WMC—were used. A set of 20 RPM problems were given to respondents, and Figure A1 shows one of these problems. This subset, of the original 60 problems, is representative of the range of difficulty present in the original. Each problem is slightly more difficult than the previous. Additionally, a sequence of Counting Span tasks, consisting of screens containing a quasi-random spatial distribution and number of colored shapes (squares, circles and triangles; blue, green and red), were presented, as shown in Figure A2. Respondents were asked to count the number of a given colored shape. At the end of the sequence (typically 3 in number), the respondent was asked to recall, in order, the numbers counted. The number of tasks given to respondents increased as the respondent answered tasks correctly and ranged between one and five.

**
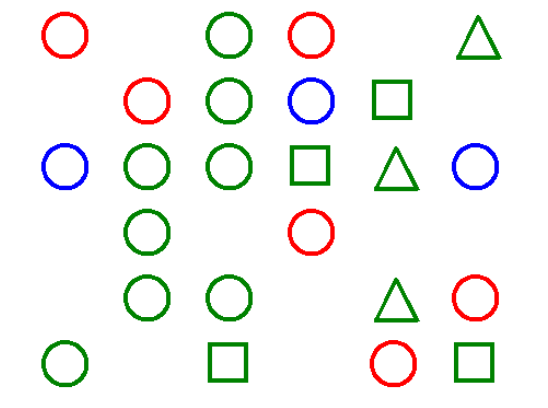
**

**Figure A2: Example of a Complex Span Counting task.**

**Study design**

Samburu was selected as the study site due to the existence of a network of relationships between researchers and community groups in south-eastern Samburu. Each of the five selected villages partners with Compassion International, Kenya in a child sponsorship program. Participation in the program was not used as a screening of study participants. A church in each village provided a site to conduct surveys and prepare and provide respondents with a free meal. Communities leaders, located within each church, used word-of-mouth recruitment of adults to participate in the study.

The five villages cover an area of approximately 35 kms^2^. The most northern village sampled was Porro and the most southerly was Kisima. The most westerly village was Loosuk. The two remaining communities were Sirata and Lare-Oibor.

Surveys took, on average, 45 minutes to complete. Prior to the first survey round, respondents were unaware that a gift of food (equivalent of $2.50) would be given to respondents. This gift acknowledged the time commitment required to complete the survey tools. It is not believed that the gift was a motivating factor for respondents to complete the survey. All respondents were also offered a basic lunch on the day they completed the survey. No explicit follow-up strategy was employed prior to round 3. Community leaders were tasked to call and remind respondents to attend the third round of data collection.

Respondents completed four tasks. The first were the cognition tasks. The ordering of these cognition tasks was randomly determined. The Discrete Choice Experiment (DCE) was completed next, followed lastly by the recall survey. The recall survey was completed using an interview style or oral question and answer. Enumerators were trained to deliver a standard, scripted, introduction and explanation for each task. Enumerators all spoke Samburu. All instructions and survey instrument were translated and available to enumerators in Swahili, Samburu and English. The DCE task and survey were both administered via tablets supporting the Open Data Kit (ODK) platform. Therefore, enumerators could toggle between languages to aid their communication. As Samburu is traditionally an oral language, enumerators found that the English and Swahili translations were most helpful as a reference. The Raven’s Progressive Matrices (RPM) and Complex Span Counting tasks were delivered via tablets that supported the e-Prime software.

Prior to each survey round enumerators were trained over two-days. Enumerators were native Samburu speakers. Each one was from one of the five sampled villages.

**Table A1: Correlation structure of variables using balanced panel data.**

|  | **ANA** | **RPM** | **Count** | **Incomee** | **Livestock Loss** | **FAO units** | **Rainfall** | **Fever** | **Ultra-poor** | **Illiterate** | **Age** | **Crop expenditure** | **Livestock expenditure** |
| --- | --- | --- | --- | --- | --- | --- | --- | --- | --- | --- | --- | --- | --- |
| **ANA** | 1.000 |  |  |  |  |  |  |  |  |  |  |  |  |
| **RPM** | -0.043 | 1.000 |  |  |  |  |  |  |  |  |  |  |  |
| **Count** | -0.067 | 0.153 | 1.000 |  |  |  |  |  |  |  |  |  |  |
| **Incomee** | -0.006 | -0.125 | -0.032 | 1.000 |  |  |  |  |  |  |  |  |  |
| **Livestock Loss** | 0.015 | -0.207 | -0.206 | 0.005 | 1.000 |  |  |  |  |  |  |  |  |
| **FAO units** | 0.031 | -0.033 | -0.050 | 0.094 | 0.118 | 1.000 |  |  |  |  |  |  |  |
| **Rainfall** | 0.059 | 0.056 | -0.167 | 0.048 | 0.052 | -0.041 | 1.000 |  |  |  |  |  |  |
| **Fever** | -0.028 | -0.054 | -0.050 | 0.109 | 0.007 | -0.084 | -0.290 | 1.000 |  |  |  |  |  |
| **Ultra-poor** | 0.080 | 0.083 | -0.143 | -0.030 | -0.025 | -0.081 | -0.060 | 0.045 | 1.000 |  |  |  |  |
| **Illiterate** | -0.013 | -0.246 | -0.223 | 0.030 | -0.096 | 0.010 | 0.024 | 0.080 | 0.066 | 1.000 |  |  |  |
| **Age** | 0.038 | -0.117 | -0.180 | 0.140 | -0.118 | 0.200 | -0.004 | 0.048 | 0.203 | 0.276 | 1.000 |  |  |
| **Crop expenditure** | -0.128 | -0.055 | 0.001 | 0.196 | 0.058 | 0.076 | -0.054 | 0.083 | -0.201 | -0.060 | 0.071 | 1.000 |  |
| **Livestock expenditure** | -0.110 | -0.043 | -0.073 | 0.158 | -0.005 | 0.181 | 0.014 | 0.018 | -0.228 | 0.072 | 0.052 | 0.290 | 1.000 |

**Table A2: Estimation of the difference between balanced and unbalanced respondents – probit.**

|  | Balanced vs Unbalanced | | |
| --- | --- | --- | --- |
|  | **Coeff.** |  | **Std. Err** |
| ANA | -0.022 |  | 0.013 |
| RPM | -0.004 |  | 0.022 |
| Count | 0.002 |  | 0.008 |
| Incomee | -0.009 |  | 0.176 |
| Livestock loss | <0.001 |  | 0.001 |
| FAO units | 0.003 |  | 0.011 |
| Rainfall | 0.016 | *** | 0.003 |
| Fever | -0.019 |  | 0.129 |
| Ultra-poor | 0.054 |  | 0.106 |
| Age | <-0.001 |  | 0.006 |
| No-School | -0.209 |  | 0.174 |
| Crop_Expenditure | <0.001 |  | <0.001 |
| Livestock_Expenditure | <0.001 | * | <0.001 |
| Constant | -2.320 | *** | 0.564 |
| N (n) | 396 (696) | | |
| AIC | 874 | | |
| BIC | 938 | | |
| Chi^2^ (p-value) | 40.29 (<0.001) | | |

Note: Statistical significance 0.05 level denoted by *, 0.01 level denoted by **, and 0.001 level denoted by ***. N represents the number of respondents and n represents number of observations. Robust standard errors used (clustered by respondent).
